# Flow Cytometric Analysis of Mediastinal Masses in Cats: A Retrospective Study

**DOI:** 10.3389/fvets.2020.00444

**Published:** 2020-08-07

**Authors:** Serena Bernardi, Valeria Martini, Stefano Perfetto, Marzia Cozzi, Stefano Comazzi

**Affiliations:** ^1^Department of Veterinary Medicine, University of Milan, Lodi, Italy; ^2^Veterinary Teaching Hospital, University of Milan, Lodi, Italy; ^3^Biessea Veterinary Laboratory, Milan, Italy

**Keywords:** cat, mediastinal masses, flow cytometry, lymphoma, thymocytes

## Abstract

Mediastinal masses occur in dogs and cats and are often investigated with cytology. However, discrimination between the two most common lesions (thymoma and lymphoma) may be challenging, especially when small/medium lymphocytes represent the prevalent population. The aim of the present study is to describe the flow cytometric aspects of mediastinal masses in cats and to assess the ability of flow cytometry (FC) to differentiate lymphoma from non-lymphomatous lesions. We retrospectively describe FC features of fine needle aspiration cytology from cats with mediastinal masses. Cases were grouped in lymphoma and non-lymphoma based on results of cytology, histopathology, PCR for antigen receptor rearrangement (PARR), clinical presentation, and follow-up. Scatter properties, positivities to CD5, CD4, CD8, CD21, CD18, and their co-expressions were recorded using a multicolour approach. Twenty cats were included, 12 lymphomas and eight non-lymphomatous cases. Forward scatter (FSC) of lymphoid cells was higher in the lymphoma group. Double positive CD4+CD8+ T-cells were the dominant population in eight out of 12 lymphomas, whereas non-lymphomatous lesions showed no dominant lymphoid population in five out of eight cases. Unlike dogs, the high prevalence of CD4+CD8+ lymphomas in cats it makes difficult to differentiate lymphoma from non-lymphomatous lesions using FC alone. FC may add interesting information to refine diagnosis in some cases, but PARR and histopathology remain mandatory to solve differential in case of expansion of small–medium sized double positive lymphoid cells.

## Introduction

The most common diagnoses for cranial mediastinal masses in cats are lymphoma and thymoma (1) followed by cystic lesions, ectopic thyroid, and other kinds of neoplasms (2).

Such lesions are commonly investigated via radiography, ultrasonography, and computer tomography (CT). All these techniques can be helpful to define the volume of the mass and the possible heterogeneity of its structure (1, 3), but they are also helpful to guide sampling procedures (fine needle aspirate cytology, FNAC), thus facilitating the investigation of its cellular origin.

A final diagnosis can be reached by cytological or histological examination, but in some cases, it may be challenging and may require further ancillary techniques. Cytology may sometimes be inconclusive because of the poor quality of samples (1) or because both lymphomas and thymomas can be composed by small/medium sized lymphocytes with minimal morphological atypia (4, 5). Thus, histopathology of tissue biopsy is often required for a definitive diagnosis. Since lymphoma and thymoma are the two most common neoplasms of mediastinal cavity in feline species, it would be desirable to distinguish them confidently in a more timely and less invasive way. In 2006, Lana et al. proposed to differentiate lymphoma and thymoma in dogs according to their antigen expression via flow cytometry (FC), since this approach had been previously used successfully in humans (6–8). This approach provided good results in canine species, with the detection of >10% CD4+CD8+ double positive small lymphocytes being highly specific for thymoma (100%). Leveraging from this paper's results, several samples from mediastinal masses also taken from cats as well have been sent to the FC service of the University of Milan in the last decade in order to better refine cytologic results. FC was preferred to other diagnostic techniques (such as PCR for antigen receptor rearrangements, PARR, or histopathology) because it is a rapid and minimally invasive test, it is not excessively expensive, and the sampling procedure is easy. FC is not intended to substitute PARR analysis or histopathology but it was likely considered useful to quickly implement the information obtained via cytology alone and to drive patient management and diagnostic pathway, considering that samples for cytology and FC may be collected in the same session with a minimal invasiveness. However, no specific studies on FC features of feline mediastinal lymphoma have been published so far.

The aim of our study was to retrospectively compare FC characteristics of lymphomas and non-lymphomatous lesions in cats with mediastinal masses and to assess whether FC could helpfully support the distinction between these two groups, thus playing a possible role in the diagnostic algorithm of mediastinal masses in cats.

## Materials and Methods

### Inclusion Criteria

The archive of the FC Service of the Veterinary Teaching Hospital of the University of Milan was retrospectively interrogated from January 2014 to November 2019, and samples of mediastinal masses in cats were extracted. Cases were retained in the study if they fulfilled the following inclusion criteria: (1) availability of FC raw data obtained on a mediastinal mass aspirate for re-analysis (CD5, CD4, CD4, CD21, and CD18; see Table 1), and (2) a final diagnosis of lymphoma or non-lymphoma based on the combination of clinical examination, imaging, cytology, histopathology, PCR for antigen receptor rearrangement (PARR), and follow-up of the patients. Clinical and clinico-pathological results were derived from the patients' medical records and eventually integrated with phone call with the referring vets about follow-up. When necessary for solving differential or confirming the final diagnosis, PARR on archive cytologic smears was performed. Cases in which a final diagnosis was not available or results were contradictory were excluded from the caseload of this study.

**Table 1:** Panel of antibodies.

**Antibody**	**Specificity**	**Clone**	**Source**
CD21-PE	B cells	CA2.1D6	Biorad, Oxford, UK
CD5-FITC	T cells	f43	SouthernBiotech, Birmingham, AL, USA
CD4-FITC	T helper lymphocytes	3-4F4	SouthernBiotech
CD8-PE	T- cytotoxic lymphocytes	fCD8	SouthernBiotech
CD18-AlexaFluor647	All leukocytes	CA1.4E9	Biorad

*Antibody panel for immuno-labeling of mediastinal masses of feline patients*.

All cats were sampled for diagnostic purposes with an informed consent of the owner. Thus, according to the guidelines of the authors' institution, a formal approval of the Ethical Committee was not required (EC decision 29 October 2012, renewed with the protocol n° 02-2016).

### Cytology

When available, cytologic smears were re-evaluated in order to confirm the FC results and to determine the prevalent cell size of lymphoid population. Cells were defined as small/medium if the nucleus diameter was less than or equal to the diameter of 2 erythrocytes (RBCs), or large if more than 2 RBCs (9). The absence of a cytologic smear or its poor quality were not considered an exclusion criterion if a final diagnosis of lymphoma or not lymphoma was confidently reached based on the combination of other clinical, clinico-pathological features, and follow-up.

### Flow Cytometry

FC was performed on tissue aspirates that were obtained from mediastinal masses and collected in a liquid transport medium (RPMI 1640).

A gross inspection of the sample was performed to assess the volume, the presence of clots, blood, or necrotic material, and a total nucleated cell count was performed either via a hematology analyser (Sysmex XT-2000iV, Sysmex, Kobe, Japan) or directly via cytometer (BriCyte E6, Mindray, Shenzhen, China) in order to assess the suitability of samples to be processed for FC analysis. The use of a vital stain (propidium iodide) to check viability and exclude dead cells and debris was not possible in most cases due to the low cellularity of most samples. Samples were processed as previously described (10). In some cases, mass samples were submitted for FC analysis together with blood or other tissues for staging purpose. Blood and effusion samples were collected into EDTA tubes, whereas fine needle aspirates were obtained from other solid tissues and suspended into tubes containing RPMI 1640. All tissues provided were processed for FC with the same operative procedure as mediastinal masses. If possible, erythrocyte lysis on samples from mediastinal masses was avoided in order to preserve poor cellularity. If gross hemodilution at visual examination or the presence of erythroid cells confounding the limits of lymphoid population at analysis were detected, erythrocytes were lysed using a solution containing ammonium chloride.

The following panel was applied to each sample, with a multicolour approach: CD5-FITC/CD21-PE/CD18-alexafluor647, CD4-FITC/CD8-PE/CD18-alexafluor647. Clone of antibodies and reactivities are listed in Table 1. Cells only were used as controls.

A minimum of 1 × 10^6^ cells was considered necessary to assess the complete panel of antibodies. If a lower cellularity was detected, the antibody panel was limited to CD4-FITC/CD8-PE/CD18-alexafluor647.

Samples were acquired with FACSCalibur flow cytometer (Becton Dickinson, San Josè, CA, USA) (since 2014 to August 2018) or with BriCyte E6 (Mindray, Shenzhen, China) (from September 2018) and analyzed with a specific software (CellQuest, Becton Dickinson or MRFlow, Mindray, respectively). A gate on lymphoid population was drawn on CD18 vs. SSC scattergram, trying to exclude debris. Percentage of positive cells falling in the lymphoid gate for each labeling, co-expressions, and median forward scatter (FSC) for lymphoid population were recorded. Positive cells were expressed as percentage out of total CD18 cells. In order to compare scatter properties of lymphoid cells obtained using two different instruments, mean FSC was then normalized for the mean FSC values obtained from circulating T lymphocytes from 10 blood samples from cats without neoplastic diseases (used as controls), in the same period, using the same instrument. Results of FSC were expressed as ratio (FSC neoplastic/FSC control). All analyses were performed by a single operator (SC) who was blinded to all information about the cases.

### PARR Analysis

When cytology, histopathology, clinical aspects, and follow-up were not conclusive to support tentative diagnosis, PARR was performed on stained slides or on archive material kept at −20°C, if they were available. In particular, PARR was performed to assess clonality in all non-lymphoma cases in which cytological slide was available and to support lymphoma diagnosis in one case (#2) which was characterized by a homogeneous expansion of medium-sized double positive T lymphocytes.

Genomic DNA was extracted and purified by using Maxwell® RSC Tissue DNA Kit (Promega, Madison, WI) following manufacturer's instructions, and the concentration of DNA in all obtained samples was evaluated by a fluorometric procedure using Quantus™ Fluorometer (Promega, Madison, WI).

T-cell receptor gamma (TCRG) and immunoglobulin heavy chain (IgH) locus were PCR-amplified using HotStarTaq Master Mix Kit (Qiagen, Valencia, CA), 100 ng of genomic DNA and primers previously designed and described (11, 12). Concentration of all primers was 10 pmol/20 μL of reaction mixture.

Amplification conditions used a 2-step modified touchdown protocol to increase specificity of the reactions. All PCR reactions were run in duplicate, and heteroduplex analysis was performed in order to separate true clonal from false-positive or pseudoclonal results (12). PCR products (10 μl) were denatured at 95°C for 10 min, then allowed to reanneal at 4°C.

Native and heteroduplex samples were separated by polyacrylamide gel electrophoresis (PAGE).

A total of 10 μl of each native and heteroduplex samples were mixed with loading buffer and loaded directly into precast 10% non-denaturing polyacrylamide Tris-Borate EDTA (TBE) gels (BioRad, Hercules, CA). Results were considered clonal in the presence of one or more reproducible bands and polyclonal with the presence of a broad smeared band or a laddering profile. Samples were considered pseudoclonal in the presence of one or more not reproducible bands (13).

Polyacrylamide gels containing both native and denatured PCR products including negative, polyclonal, and clonal controls were run in TBE at 150 V for 2 h.

The gels were stained with ethidium bromide and visualized under UV light.

### Serology for Retroviral Infections

Data on serological tests for Feline Leukemia Virus (FeLV) and Feline Immunodeficiency Virus (FIV) were not specifically performed in this research but, if available, they were collected from the referral clinician.

### Statistical Analysis

All data were recorded in an electronic datasheet and descriptive statistics were calculated (including mean, standard deviation, median). Normal distribution of continuous variables was checked via Shapiro-Wilk test.

A chi-squared Pearson's test was performed to detect possible difference in sex distribution between lymphoma and non-lymphoma groups, whereas possible difference in median age between the two groups was checked *via* Mann-Whitney test.

The latter test was also performed to assess possible differences in median FSC values between the two groups. Conversely, differences in mean percentage of CD4+CD8+ double positive cells were investigated via Student *t*-test for independent samples. Homoscedasticity was checked via Levene's test. ROC curves were drawn for FSC values and CD4+CD8+ percentages, and the coordinates were analyzed to identify a cutoff suitable to discriminate between lymphomas and non-lymphomas. The values showing the best compromise between sensitivity and specificity were chosen as cutoffs.

All statistical analyses were performed with SPSS 20.0 software, setting the significance level at *p* ≤ 0.05 for all tests.

## Results

### Caseload

Twenty-seven cases of feline mediastinal masses were retrieved in the FC database, from 2014 to 2019. Of these, three samples were excluded because they were acellular, and four were excluded because a final diagnosis could not be reached. Hence, 20 cases were included in the study, including 12 (60%) lymphomas and 8 (40%) non-lymphomatous lesions.

Cats with lymphoma were significantly younger than those with other diseases (*P* = 0.002). Median age for cats with lymphoma was 4 yo while median age for cats without lymphoma was 9.5 yo. No gender predilection was found for both groups of lesions. Data on signalment and clinico-pathological features (including serology for retroviral diseases) and criteria used for final diagnosis of lymphoma vs. non-lymphoma in the 20 cats are described in Table 2.

**Table 2:** Caseload.

**Case#**	**Patient**	**Gender**	**Age**	**FIV/FeLV status**	**Criteria for diagnosis**	**Final diagnosis**
1	DSH	Fn	3 y, 9 m	Unknown	Histopathology	LYMPHOMA DP
2	DSH	Fn	4 y	Unknown	PARR (monoclonal)	LYMPHOMA DP
3	BSH	Mc	9 m	FELV neg,	Cytology, histopathology	LYMPHOMA DP
				FIV neg		
4	DSH	Fn	4 y	FELV pos,	Cytology, imaging, follow-up	LYMPHOMA DP
				FIV neg		
5	DSH	Fn	8 y	Unknown	Cytology, FC pseudoclonality (homogeneous population of CD4 + T cells)	LYMPHOMA T4
6	DSH	Mc	8 y	FELV neg,	Cytology, FC pseudoclonality (homogeneous population of CD4 + T cells)	LYMPHOMA DP
				FIV neg		
7	SBI	Mc	4 y	Unknown	Cytology, FC pseudoclonality (homogeneous population of CD4-CD8- T cells)	LYMPHOMA DN
8	DSH	Mc	2 y	Unknown	Cytology, neoplastic cells in PB, lymph nodes, thoracic effusion	LYMPHOMA DP
9	DSH	Fn	2 y	Unknown	Cytology, FC pseudoclonality (prevalent population of CD5-CD4 + cells)	LYMPHOMA T4
10	Unknown	Fn	8 y	Unknown	FC pseudoclonality (prevalent population of CD5-CD4 + cells)	LYMPHOMA T4
11	DSH	M	2 y	Unknown	Cytology, neoplastic cells in PB and thoracic effusion	LYMPHOMA DP
12	DSH	Mc	4 y	Unknown	Neoplastic cells in lymph nodes	LYMPHOMA DP
13	DSH	Fn	10 y	Unknown	Imaging, follow-up	NON-LYMPHOMA
14	DSH	Mc	5 y	Unknown	Cytology, PARR (polyclonal)	NON-LYMPHOMA
15	DSH	Fn	12 y	Unknown	Cytology, PARR (polyclonal)	NON-LYMPHOMA
16	Unknown	Unknown	6 y	Unknown	Cytology, PARR (polyclonal)	NON-LYMPHOMA
17	DSH	Fn	10 y	Unknown	Cytology, PARR (polyclonal)	NON-LYMPHOMA
18	DSH	Fn	9 y	Unknown	Cytology, PARR (polyclonal)	NON-LYMPHOMA
19	DSH	Mc	8 y	Unknown	Cytology, PARR (polyclonal)	NON-LYMPHOMA
20	DSH	Fs	15 yo	Unknown	Paraneoplastic exfoliative dermatitis, imaging, follow-up	NON-LYMPHOMA

*Signalment and retroviral status and criteria used for diagnosis in 20 cats with mediastinal masses*.

*DSH, domestic shorthair; BSH, British shorthair; SBI, Sacred Birman; FN, female neutered; MC, male castrated; M, male; y, year/s; m, month/s PB, peripheral blood; T4, CD4 positive; DP, double positive CD4+CD8+; DN, double negative CD4-CD8-; T4 CD4+CD8-; pos, positive; neg, negative*.

Serology for retroviral diseases was available just in three out of 20 cases. In only one case (CD4+CD8+ double positive lymphoma) it scored positive for FeLV while FIV was negative in all three cases.

### Cell Sizes in Cytology and FC

Results of evaluation of cell size via cytology and FC are expressed in Table 3. Lymphoma cases showed a significantly (*P* = 0.001) higher FSC (mean = 1.40, standard deviation = 0.17, median = 1.38, range = 1.19–1.85) in comparison with non-lymphomatous masses (mean = 1.03, standard deviation = 0.19, median = 0.96, range = 0.83–1.34). Cytological evaluation of cell size confirmed these results with lymphoma cases showing small/medium size in five cases out of eight smears available while non-lymphoma cases were all characterized by small/medium cells (six cases out of six smears available). A cut-off value of 1.30 was elected as the best to discriminate lymphoma from non-lymphomatous lesions with 83% sensitivity and 88% specificity.

**Table 3:** Lymphoid populations.

**Case#**	**Final diagnosis**	**CD5**	**CD4**	**CD8**	**dp**	**dn**	**CD21**	**FSC ratio**	**Cytologic size**
1	LYMPHOMA DP	98, 0	13, 1	12, 7	67, 7	4, 5	<1	1, 37	–
2	LYMPHOMA DP	99, 4	<1	<1	99, 3	<1	<1	1, 52	–
3	LYMPHOMA DP	99, 7	<1	<1	99, 8	<1	<1	1, 2	Small/medium
4	LYMPHOMA DP	99, 8	10, 7	<1	89, 1	<1	<1	1, 38	Small/medium
5	LYMPHOMA T4	98, 7	88, 6	<1	11, 0	<1	<1	1, 44	Large
6	LYMPHOMA DP	100, 0	25, 8	<1	73, 1	<1	<1	1, 43	Small/medium
7	LYMPHOMA DN	94, 6	11, 8	8, 6	1, 34	72, 9	<1	1, 85	Large
8	LYMPHOMA DP	ND	1, 6	<1	96, 7	<1	<1	1, 37	Small/medium
9	LYMPHOMA T4	32, 2	60, 8	<1	<1	<1	<1	1, 39	Large
10	LYMPHOMA T4	9, 7	87, 0	1, 9	1, 2	<1	<1	1, 19	–
11	LYMPHOMA DP	94, 3	14, 3	3, 1	82, 0	<1	<1	1, 32	Small/medium
12	LYMPHOMA DP	98, 2	1, 2	16, 9	81, 4	<1	<1	1, 34	–
13	NON-LYMPHOMA	73, 9	8, 1	6, 1	78, 0	<1	<1	0, 88	–
14	NON-LYMPHOMA	84, 3	24, 1	30, 7	2, 7	26, 8	<1	0, 94	Small/medium
15	NON-LYMPHOMA	92, 2	22, 8	43, 8	17, 9	7, 7	1, 9	1, 31	Small/medium
16	NON-LYMPHOMA	86, 8	18, 2	28, 1	45, 5	<1	<1	1, 34	Small/medium
17	NON-LYMPHOMA	84, 7	9, 9	8, 1	51, 2	15, 6	<1	0, 98	Small/medium
18	NON-LYMPHOMA	97, 7	8, 1	5, 6	78, 8	5, 2	<1	0, 83	Small/medium
19	NON-LYMPHOMA	79, 7	21, 19	27, 9	24, 4	6, 2	<1	1, 04	Small/medium
20	NON-LYMPHOMA	90, 4	5, 8	8, 5	73, 1	12, 1	<1	0, 93	–

*Percentages of positive cells in lymphoid population from 20 cats with mediastinal masses*.

*Percentages are expressed out of all CD18 positive cells and were calculated just in the population of cells with low side scatter properties (likely lymphoid in origin). Residual population of cells, when present, is composed by cells with high side scatter, which are negative to all the markers, likely granulocytes, monocytes and macrophages (not reported in the table). T4, CD4 positive; DP, double positive CD4+CD8+; DN, double negative CD4-CD8-*.

### Lymphoid Cell Subpopulations

Composition of lymphoid cell populations from cats with lymphoma and non-lymphoma are shown in Table 3.

**Figure 1:**
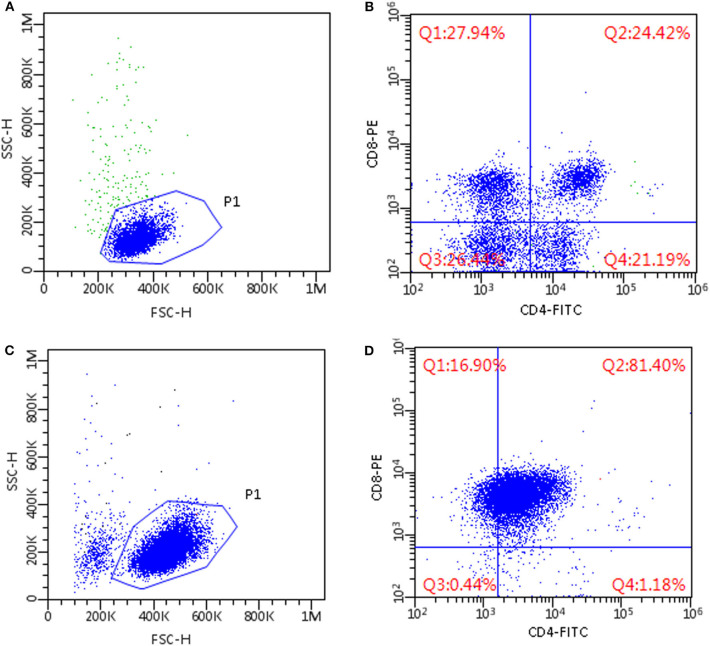
Flow cytometric scattergrams of mediastinal masses in two cats. **(A,C)** Morphological scattergrams, a gate (P1) was set to exclude debris and disrupted cells. **(B,D)** Only P1 cells are shown, based on CD4 and CD8 staining. Fluorescence discriminators were set based on the level of fluorescence of unstained cells from the same sample. **(A,B)** Non-lymphomatous lesion; cells are small-sized and composed of mixed subpopulations of T-cells, including CD4+CD8+ double-positive cells (**B**, upper right quadrant). **(C,D)** CD4+CD8+ double positive T-cell lymphoma; cells are medium sized and a dominant population of CD4+CD8+ double positive cells is present (**D**, upper right quadrant).

In all lymphoma cases, a prevalent lymphocyte subpopulation was found, representing more than 60% of CD18+ lymphoid cells, whereas other residual lymphocyte subsets were poorly represented (Figures 1C,D).

The most commonly established immunophenotype of lymphomas was CD4+CD8+ double positive (eight cases, 67%); three cases (25%) showed a CD4+ T-cell phenotype and one case (8%) showed a CD4-CD8- double negative T-cell phenotype. In two of the 3 CD4+ lymphomas (#9 and #10, 66.7%), an aberrant phenotype was found, with 60.8 and 87% of cells, respectively, staining positive for CD4, but mostly negative for CD5.

In 1 case (#8) the sample was processed with a reduced panel because of poor cellularity, which allowed labeling just with anti-CD4 and -CD8 antibodies, revealing 96.7% double positive cells; these cells were also detected in high percentages in thoracic fluid, thoracic lymph node, and blood of the same animal.

In non-lymphomatous masses FC immunophenotyping revealed >79% of CD18+ lymphoid cells in the samples to be T lymphocytes expressing CD5. No dominant lymphoid population could be identified in five cases (Figures 1A,B), whereas the remaining samples made up for 78.8% and 73.1 by CD4+CD8+ double positive cells. In particular, CD4+CD8+ double positive cells were >40% in five out of eight cases and <10% in one case.

The percentage of CD4+CD8+ cells was statistically higher in double positive lymphoma (mean = 85.9, s.d. = 12.3, median = 85.5, range 67.7–99.8) than in non-lymphoma samples (mean = 46.5, s.d. = 29.2, median = 48.4, range 2.7–78.8) (*P* = 0.006). A cut-off value of 75% was elected as the best to discriminate double positive lymphoma from non-lymphoma with 75% sensitivity and 75% specificity.

### PARR Analysis

PARR was performed in one case of lymphoma (#2), in which final diagnosis was challenging, and in all six cases of non-lymphoma (cases #14–19) in which a cytological smear adequate for DNA extraction was available. Monoclonal spike was detected in case #2 that allowed it to be included in the lymphoma group. All the other cases show a polyclonal result, thus confirming the suspicion of non-lymphoma.

## Discussion

Mediastinal lymphoma is quite common in young and FeLV positive cats (14, 15). Results of our caseload support that the median age of cats with mediastinal lymphoma is lower than that of cats with non-lymphomatous mediastinal masses. Thymoma is by far the most frequent non-lymphoid tumor occurring in mediastinal space mainly in middle age to old cats (3).

Although the two diseases are different in terms of origin and pathogenesis they may be similar in terms of clinical symptoms, imaging, and cytological appearance. In both cases small to medium lymphocytes are the prevalent population collected at FNAC since neoplastic epithelial cells (in thymoma) may sometimes exfoliate poorly because of their high cohesivity. In addition, the morphological characteristics of lymphoid population of thymus may be difficult to differentiate from that of a small cell lymphoma since cells may exhibit signs of immaturity and may appear undifferentiated. In one retrospective study in the dog, only seven of 17 mediastinal masses could be definitively diagnosed by cytology (16) and this appears likely in cats as well. In contrast, the two diseases tend to exhibit a different prognosis and to benefit from different therapeutic approach, with chemotherapy the preferred option for lymphoma while surgery and/or radiotherapy are often suggested for thymoma (17, 18). Histopathologic biopsy may help to reach a definitive diagnosis but tru-cut biopsy, which is the easiest technique to sample mediastinal masses, may sometimes provide inconclusive results while surgical biopsies are often considered invasive procedures. PARR may also help to differentiate lymphoma and non-lymphoma. To the best of our knowledge no specific papers on the use of PARR for differentiating lymphoma vs. thymoma in cats are available, but results of PARR analysis in a cohort of 13 dogs with thymoma was recently published and found a polyclonal result in 12 cases while T-cell clonality was detected in one case (19). In the cat, PARR and FC were used to confirm the origin from an ectopic thymoma in a cat with cervical mass from ectopic thymoma (20). In this case, however, FC results did not allow to discriminate a T-cell double positive lymphoma from thymoma, and only PARR and histopathology allowed for a final diagnosis. In spite of the advantages of PARR for detecting lymphoid clonal expansion, some false negative results may also occur (21), and PARR is time consuming. Therefore, results may be available several days after sampling, which is a limiting factor in clinical conditions. Moreover, PARR does not allow important morphological evaluation; moreover, it should be complementary to other assay and it should not be used to determine the detailed immunophenotype of T-cell neoplasia since cross lineage results occur (21).

In 2006, Lana and collaborators tried to apply FC characterization of lymphoid population from mediastinal masses in dogs and found that the presence of >10% CD4+CD8+ thymocytes is highly suggestive of non-lymphomatous lesions. Here we describe the application of FC to feline mediastinal masses, highlighting some differences with the canine counterpart that decrease the potential diagnostic usefulness in feline species.

FSC properties and cell size in cytology confirmed that non-lymphomatous masses are composed by cells similar to T lymphocytes found in peripheral blood from healthy cats, whereas neoplastic cells in lymphomas tend to be greater in size. Cellular size at microscopical evaluation and FSC properties are not equivalent, and some discrepancies may occur as we can see in the available caseload. The first is based on the size of nucleus while the latter is referred to the volume of the cells. The latter is probably more accurate, due to the high number of cells evaluated and the more objective evaluation.

Double positive CD4+CD8+ T-cell is by far the most represented phenotype in feline mediastinal lymphomas in our caseload (eight cases, 67% of lymphoma cases), whereas it is relatively rare in dogs and most mediastinal lymphoma exhibit CD4+ T-cell phenotype (5). Because of this high percentage of double positive lymphoma samples in feline species, the cut-off value of 10% applied in dogs with a 100% specificity is not valid for cats.

Despite this gross difference between the two species to reduce the diagnostic role of FC in diagnosis of feline mediastinal mass, some suggestions may derive from our results, which should be taken into account when dealing with FC analysis of mediastinal mass aspirates in cats: (1) FSC properties help to support in a more objective way the cytologic estimation of cell size being generally smaller in non-lymphoma samples than in lymphoma; (2) lymphoma is highly probable if the dominant population is large-sized, irrespective of the phenotype; (3) the presence of a dominant lymphoid population other than double positive CD4+CD8+ cells or of cells with aberrant phenotype is strongly suggestive of lymphoma; (4) a mixed population of T lymphoid cells composed by CD4+, CD8+, double positive CD4+CD8+, and double negative CD4-CD8- lymphocytes is strongly suggestive of non-lymphomatous lesions; (5) a prevalent population of double positive lymphoid cells may be encountered in both lymphomas and non-lymphomatous lesions. In this case, further clinico-pathological features may help to reach a final diagnosis, such as a larger size of lymphoid cells (FSC ratio higher than 1.30) and the presence of cells with the same FC features in other tissues from the same animal (both being supporting of lymphoma). In addition, although not definitive, FC may suggest double positive lymphoma if a homogeneous population (more than 75%) of double positive lymphocytes is found. PARR could help to confirm clonality in lymphoma and histopathology may lead to a definitive diagnosis, although some cases remain challenging, mainly if a tru-cut biopsy has been performed.

The present study has some limitations. First the low number of cases analyzed and its retrospective nature limit the power of the results and the potential application in a clinical setup. Second, histopathology was not available in most cases and a good quality cytology smear was not available for some of the samples. This is why we preferred to split our cases in lymphoma vs. non-lymphoma cases rather than lymphoma vs. thymoma. Moreover, residual cytological smears for PARR were not available in all the cases. Due to this lack of consistency among diagnostic tests available, final diagnosis was obtained by putting together clinical signs reported by the referral clinician (including imaging and presence of paraneoplastic diseases), by cytology, histopathology, and PARR results, by the presence of pseudoclonality via FC, by the presence of neoplastic cells in other tissues than mediastinal mass and by monitoring the follow-up (including response to chemotherapy, radiotherapy, or surgery). As an example, in case #4 the good and rapid response to CHOP-based chemotherapy confirmed the cytological suspect of lymphoma in a young FeLV positive cat. In contrast, in case #13 the cat was treated by CHOP and 12 fractions of radiotherapy with an initial reduction of the mass followed by a stable disease. After that, clinicians reported that in-house cytology was repeated, showing the presence of several epithelial clusters of neoplastic cells in the context of a lymphocyte rich FNAC, thus supporting the diagnosis of thymoma. Cases in which final diagnosis was not confidently reached were excluded from the present caseload.

Finally, FC data were obtained using two different instruments during the study period due to the acquisition of new instrumentation in the lab. The use of different instruments, although adequately controlled and standardized, prevents the comparison between scatter properties and fluorescence intensity but minimally affects results in terms of percentage of positive cells. We tried to overcome this issue by comparing the size of lymphoid cells in the masses with the size of non-neoplastic lymphocytes in cats without neoplasia analyzed with the same instrument in the same period. This may offer a better opportunity to compare results between the two different instruments as well as to provide reproducibility for other institutions. A prospective study on a larger caseload using a standardized approach and a single instrument may be useful to confirm our results.

## Conclusion

In conclusion, FC in cats is not helpful to confirm mediastinal lymphoma like it is in dogs, even though a suspecion of lymphoma may be considered in cats if lymphoid cells are large-sized cells; a highly prevalent population of cells other than double positive CD4+CD8+ lymphocytes or an aberrant phenotypes are detected. FC may also provide some useful information to support the diagnostic process in other cases. Unfortunately, double positive mediastinal lymphoma is frequent in cats, thus limiting the diagnostic power of FC compared to dogs. A comprehensive evaluation of all anamnestic and clinico-pathological features may help in some cases, but PARR and histopathology are strongly suggested to reach a final diagnosis in case of expansion of small-medium sized double positive lymphoid cells.

## Data Availability Statement

The raw data supporting the conclusions of this article will be made available by the authors, without undue reservation.

## Ethics Statement

Ethical review and approval was not required for the animal study because all cats were sampled for diagnostic purposes with an informed consent of the owner. Thus, according to the guidelines of the authors' Institution, a formal approval of the Ethical Committee was not required (EC decision 29 October 2012, renewed with the protocol n° 02-2016). Written informed consent was obtained from the owners for the participation of their animals in this study.

## Author Contributions

SC planned the research. SB, SC, and VM wrote the manuscript. VM performed statistical analysis. SB, VM, and MC performed flow cytometry analysis. SP performed PARR analysis. All authors contributed to discussion of results and correction of manuscript drafts. All authors contributed to the article and approved the submitted version.

## Conflict of Interest

The reviewer BR declared a past co-authorship with several of the authors SC and MC to the handling editor. The remaining authors declare that the research was conducted in the absence of any commercial or financial relationships that could be construed as a potential conflict of interest.
